# Coordinating human-robot collaboration by EEG-based human intention prediction and vigilance control

**DOI:** 10.3389/fnbot.2022.1068274

**Published:** 2022-12-01

**Authors:** Jianzhi Lyu, Alexander Maýe, Michael Görner, Philipp Ruppel, Andreas K. Engel, Jianwei Zhang

**Affiliations:** ^1^TAMS Group, Department of Informatics, University of Hamburg, Hamburg, Germany; ^2^Department of Neurophysiology and Pathophysiology, University Medical Center Hamburg-Eppendorf, Hamburg, Germany

**Keywords:** human-robot collaboration, brain-computer interface, intention prediction, collision avoidance, trajectory optimization

## Abstract

In human-robot collaboration scenarios with shared workspaces, a highly desired performance boost is offset by high requirements for human safety, limiting speed and torque of the robot drives to levels which cannot harm the human body. Especially for complex tasks with flexible human behavior, it becomes vital to maintain safe working distances and coordinate tasks efficiently. An established approach in this regard is reactive servo in response to the current human pose. However, such an approach does not exploit expectations of the human's behavior and can therefore fail to react to fast human motions in time. To adapt the robot's behavior as soon as possible, predicting human intention early becomes a factor which is vital but hard to achieve. Here, we employ a recently developed type of brain-computer interface (BCI) which can detect the focus of the human's overt attention as a predictor for impending action. In contrast to other types of BCI, direct projection of stimuli onto the workspace facilitates a seamless integration in workflows. Moreover, we demonstrate how the signal-to-noise ratio of the brain response can be used to adjust the velocity of the robot movements to the vigilance or alertness level of the human. Analyzing this adaptive system with respect to performance and safety margins in a physical robot experiment, we found the proposed method could improve both collaboration efficiency and safety distance.

## 1. Introduction

Modern industrial production facilities include a variety of manipulation tasks, many of which have to be performed by robots and require super-human precision. Others require human-level dexterity and can (as of now) only be handled by human workers. When these task domains become entwined for complex assembly, the concepts of human-robot collaboration (HRC) and shared workspaces enter the picture to make use of both robot automation and human intelligence (Castro et al., 2021). However, especially in tight workspaces, HRC approaches must fulfill the strict requirements for human safety. Human motion is often fast and diverse, whereas robots should remain relatively slow in HRC scenarios to be predictable and safe. Thus, it becomes challenging to adjust the robot's behavior in time to coordinate with the human's movements.

For physical HRC tasks, e.g., handover or collaborative carrying, the interaction forces between the robot and the human constitute the most important aspect of safety and are usually handled by high-frequency impedance controllers (Ficuciello et al., 2015; Agravante et al., 2019; Stouraitis et al., 2020). In contrast, this paper focuses on a collaboration scenario without physical interaction and instead explores how to strike an efficient balance between working distances and task performance. The key to achieve this goal is to endow robots with the ability to not only monitor but also predict the human partner's movements or general task plan. Several studies have investigated methods to monitor or predict human behavior. In Qi and Su (2022), a multimodal network was proposed to monitor human activities and health states. As to human behavior prediction, one approach used time series classification to predict human reaching targets (Pérez-D'Arpino and Shah, 2015). The human palm trajectory was modeled as a multivariate Gaussian distribution, and the human's intention was estimated by conditioning on observed partial trajectories. Another approach employed Gaussian Mixture Model (GMM) to classify human arm trajectories (Luo et al., 2018; Park et al., 2019). It classified human reaching actions through an offline learning method and could generate collision-free and smooth trajectories for a robot arm. Human motion was implicitly modeled through a reward function of hand-target distance and hand velocity (Cheng et al., 2020; Zhao et al., 2020). In a more related work (Lyu et al., 2022), a framework was proposed to combine human trajectory prediction, intention estimation, and robot trajectory optimization to achieve efficient and collision-free HRC. Other methods included gaze information in HRC scenarios. Vision and arm motion data were fused for human reach target prediction (Ravichandar et al., 2018). The proposed gaze estimation method was based on external camera data and failed when the human face was occluded. Gaze information from an eye tracker was considered to evaluate whether the human got distracted by the robot's actions (Cini et al., 2021). In Trick et al. (2019), gaze, gesture, and speech information were fused in a Bayesian framework to reduce intention recognition uncertainty.

We think that multidisciplinary research can provide alternative information channels between the robot and the human and help improve HRC (Kragic and Sandamirskaya, 2021). To this end, we investigate the use of brain-computer interfaces (BCIs) in combination with online trajectory optimization for safer and more efficient HRC. Robot control is a prominent application area for BCIs. Applications can be broadly grouped into human intention prediction for collaboration and direct or high-level robot control for teleoperation. The time point of a movement onset can be predicted from the readiness potential, a conspicuous deflection in the EEG signal which starts about 500 ms before the movement. This signal can be used to predict whether the human will move the left or right arm, and the speed of the robot can be adjusted according to whether or not the human is in the shared workspace (Cooper et al., 2020; Buerkle et al., 2021). Movement-related cortical potentials, recorded by an EEG amplifier around the time of movement onset, have been shown to be informative about the upcoming grasp action, e.g., palmar, pinch, etc. (Xu et al., 2021). Motor imagery is another prominent BCI paradigm for communicating the human's intention. Imagining to move one's hands, feet or the tongue generates different patterns in the EEG topographies, and impressive classification accuracy of these patterns by deep neural networks have been achieved (Zhang et al., 2019; Huang et al., 2020). A larger number of intentions can be encoded by mapping them to keys of a BCI spelling application. For example, a P300 BCI has been used to control an assistive robot arm and a mobile robot (Song et al., 2020).

Another BCI paradigm makes use of the brain's response when the human looks at flickering visual stimuli (steady-state visual evoked response, SSVEP), and it has been used to select targets in a pick-and-place scenario (Chen et al., 2019). Typical P300 and SSVEP BCIs present the user interface on a computer screen or LED panel. Hence a human operator has to continuously switch gaze between the workspace, where he plans the next operation, and the BCI interface to issue the corresponding command for the robot (Chen et al., 2018, 2019; Song et al., 2020). Augmented reality environments have been used to overlay the BCI interface with a camera view of the workspace, but they require the human to wear virtual reality equipment, and corresponding studies are scarce (Liu et al., 2019; Ke et al., 2020). To address the downsides of these SSVEP BCIs, we employ a recently developed variant that can recognize the gaze direction relative to a single flicker stimulus (Maye et al., 2017). For this spatially-coded SSVEP BCI, the user interface can easily be projected on the workspace, and the operator can control the BCI by gazing at dedicated locations in the workspace. As humans tend to look at the location where they are going to deploy an action (Land et al., 1999; Johansson et al., 2001), we suggest that detecting the gaze direction enables seamless integration of the intention prediction in the human's workflow.

In addition to this overt attention detection, we decode and monitor the vigilance or attention level of the operator. EEG-based methods for vigilance monitoring have been investigated for decades, and the literature abounds with corresponding methods (reviewed in Stancin et al., 2021). Using an SSVEP BCI for intention prediction suggests looking at SSVEP-based vigilance monitoring methods. It has been known since the end of the 1980s that attention can modulate the amplitude of visual evoked responses (Mangun and Hillyard, 1988). For SSVEP BCIs, it has been shown that fatigue and fading attention is associated with reduced amplitude and signal-to-noise ratio (SNR) of the SSVEP response (Cao et al., 2014). Other studies found an inverse relationship between attention and SSVEP amplitude (Silberstein et al., 1990), and the actual association may depend on the brain network that responds to the stimulus frequency (Ding et al., 2005; Gulbinaite et al., 2019). Here, we consider vigilance as a dynamic process which is not only influenced by the human's ability to maintain a stable attention level or fatigue but also by the interaction with the robot. Therefore we monitor vigilance changes on a sub-minute scale (Jung et al., 1997) and take the SSVEP SNR as an index of the operator's vigilance which can modulate the robot's behavior.

Most studies on BCI methods for HRC focus on the development of the BCI component (Chen et al., 2018, 2019) or evaluate the interaction in simulation (Buerkle et al., 2021). Here, we demonstrate methods for integrating BCI output as well as human arm tracking data with the robot controller in a physical setup. To this end, we integrated the reach target prediction and vigilance monitoring from the BCI with an online trajectory optimizer. The robot's current workspace target was adjusted online according to the BCI-based intention predictions, and the robot's operating velocity was modulated according to the operator's vigilance level. Human arm positions were tracked online and used by a trajectory optimization algorithm for collision avoidance, ensuring human safety. The system was evaluated on seventeen participants, comparing three conditions in which the robot controller received information about the target either after the operator started to move the arm (arm tracking), before moving the arm (BCI), or adjusted the robot's velocity according to the operator's vigilance in addition to the BCI-based target prediction. Our results suggest that the proposed BCI and vigilance monitoring strategy can improve both HRC efficiency and safety at the same time.

Given the above, the key contributions of this work are as follows:

1. To our best knowledge, this is the first attempt to investigate the use of a BCI in combination with online trajectory optimization for HRC in a narrow shared worksapce.
2. We employed a recently developed BCI paradigm that can recognize the gaze direction relative to a single flicker stimulus to predict human intention. The SSVEP SNR was used as an indicator of the human's vigilance to modulate the robot's behavior.
3. We demonstrated the method of integrating BCI output as well as human arm tracking data with the robot controller.

## 2. Materials and methods

### 2.1. Setup and task description

This study targets scenarios with non-physical collaboration. In such setups, the robot and a human work at multiple locations in the same workspace, but they are never assigned the exact same work location at the same time. Such tasks include component allotment (Pérez-D'Arpino and Shah, 2015; Li and Shah, 2019; Park et al., 2019; Unhelkar et al., 2020), collaborative assembly (Cheng et al., 2020; Zhao et al., 2020), and daily food preparation (Unhelkar et al., 2020). We consider a similar scenario about collaborative screw assembly. In this scenario, there are five products to be screwed in five locations. The human will put the screws into the screw holes of the products, and the robot needs to get the screws in. In order not to interfere with humans, the robot must work on different products with screws from the one the humans are working on. To simplify, we simulate this scenario using stand-in touch gestures and define five target locations, marked by colored blocks, between the human and the robot.

**Figure 1:**
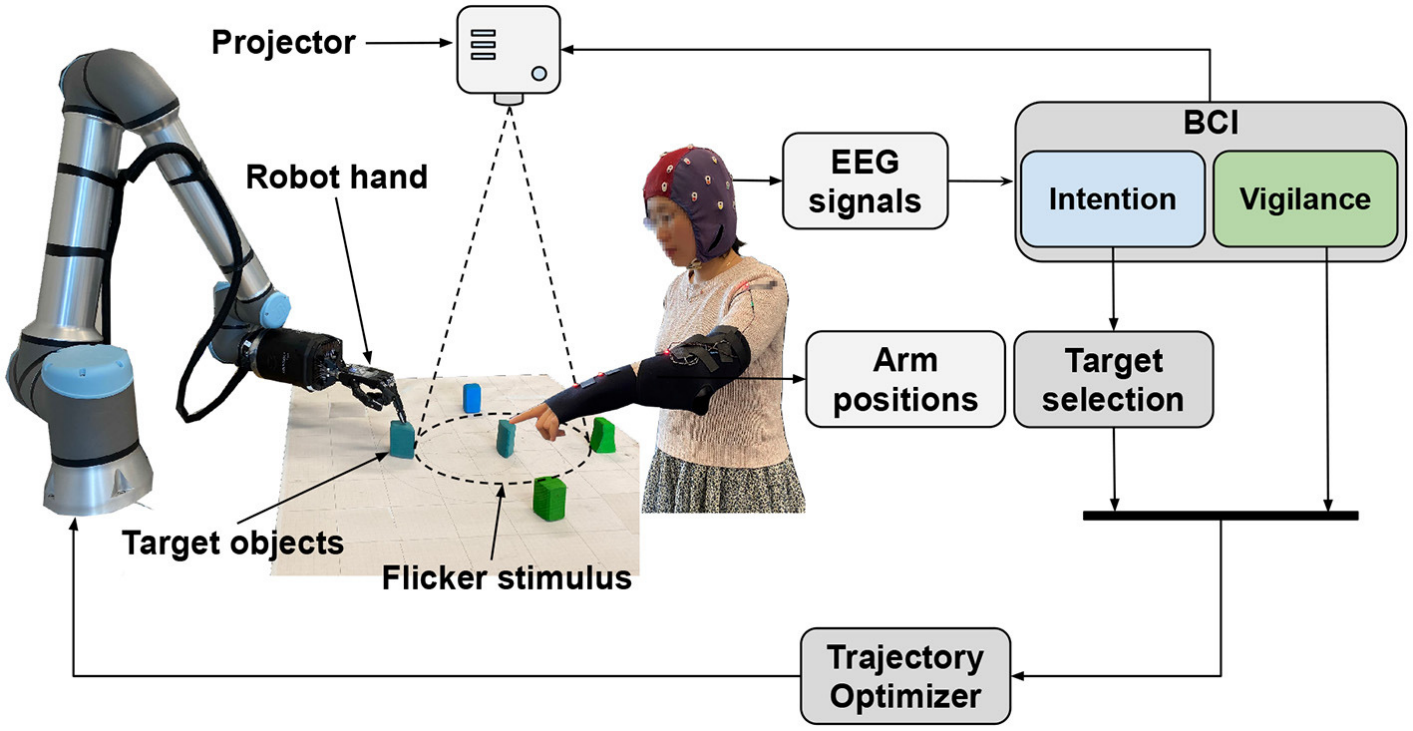
The proposed HRC framework.

We used a UR10e robot arm with a Shadow C6 dexterous left hand. The architecture of the proposed HRC framework is shown in Figure 1.

The human and the robot touched the individual blocks with their index finger tips. At each target location, they hovered the hand for 1.5 s above the block and actuated a simple finger motion to touch its top. The robot had to avoid contact with the human's arm and needed to adjust its reach target location accordingly. In addition, the robot's arm velocity was modulated by the vigilance level of the human.

### 2.2. Participants

Seventeen subjects participated in the study; five of them were female. They were between 17 and 40 years old (mean: 27.82 years). All of them had normal vision and were free of neurological and ophthalmological disorders. All but two participants declared to be right-handed; the two left-handers stated that their right hand would be equally skilled with regard to the described task. Eight participants were external to the associated institutions and received financial compensation.

Before the experiment, the purpose and content of the study were explained to the participants. Safety procedures were pointed out including velocity and force limits of the robot arm as well as a foot pedal and emergence stop controlled by the experimenter. The participants were informed that they had the right to quit the experiment at any time without justification. A consent form was provided and explained to the participants in detail before the experiments.

### 2.3. Experimental conditions

The study was comprised of three conditions, preceded by an initial training session to calibrate the BCI. The order of the conditions was randomized and counterbalanced across participants.

In the training session, the robot hand approached the targets in a fixed order, tipping each one as described above. The participants were instructed to stand still in front of the table and gaze at the target positions that were cued by the BCI (see below). The recorded EEG signals were used to train the classifier of the BCI.

In the arm-tracking condition (AT), the online robot controller relied solely on arm tracking data for safe robot control. For each time step, the target location that was closest to the participant's palm was taken to be the current reach goal of the participant. The robot's velocity limits were constant. This condition served as a baseline for evaluating the two BCI-enabled conditions.

The BCI condition provided the robot controller with the target location that the participant was gazing at before commencing the reach gesture. The operator instructions and robot control method were the same as for the AT condition.

The third condition (BCI+VCV) utilized the BCI target prediction but additionally considered the current estimation of the participant's vigilance for controlling the robot's velocity (vigilance-controlled velocity). Following the hypothesis that a more vigilant participant can interact safely with a faster robot partner, the estimated vigilance was used to adjust the robot's joint velocity limits within a margin.

In the breaks between sessions, participants were encouraged to improve the classification accuracy of the BCI in the next session by increasing their visual attention to the target locations and suppressing any distracting thoughts.

### 2.4. Brain-computer interface

A recently developed, spatially-coded SSVEP BCI was employed to detect the human's gaze direction. In contrast to conventional frequency-coded SSVEP BCIs in which targets are defined by different frequencies and/or phases of several flicker-stimuli, it requires only a single flicker-stimulus and identifies targets from the location of this stimulus in the visual field of the operator. Preliminary results suggest that this approach may reduce visual strain from operating the BCI in addition to simplifying its stimulation setup. A detailed description of the paradigm and a comprehensive analysis of its performance can be found in Maye et al. (2017); here, we explain its adaptation to the HRC scenario.

**Figure 2:**
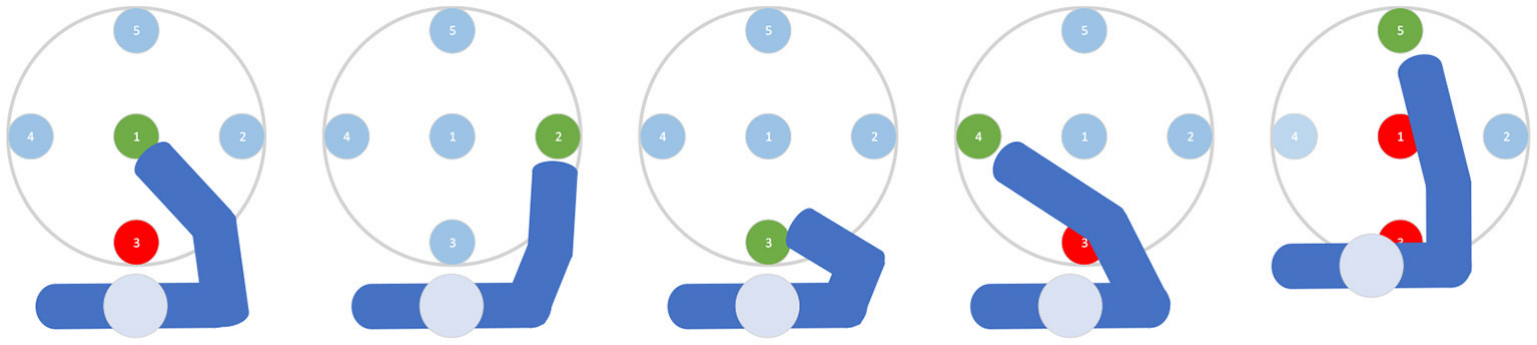
Relationship between valid target locations for the robot (light blue targets) given an intended human target location (green). Red targets are blocked by the human arm.

Reach target locations can be decoded from brain activity by their position in relation to a flicker stimulus. This visual flicker elicits a standing wave of steady-state evoked potentials (SSVEPs) in the occipital cortex at the flicker frequency, and the topographic distribution of this response across the scalp can be used to infer the human's gaze direction. Since EEG topographies show substantial variability across the population, the approach works best when for each user samples are recorded in a training session and used for classifying data in the application phase. EEG data were recorded from 32 electrodes placed on the scalp according to the 10-20 system and connected to an ActiveTwo amplifier (BioSemi instrumentation, Amsterdam, The Netherlands). This system employs active electrodes and common mode rejection to reduce artifacts from external electromagnetic fields, making it suitable for application in environments with common levels of electromagnetic noise. Apart from band-pass filtering, no other measures were taken to eliminate artifacts in the EEG data. The flicker stimulus in our setup was a white disc appearing and disappearing with a frequency of 15 Hz. The disc was projected through a Toshiba TDP P9 overhead projector on the workspace and had a diameter of 40 cm. Five target locations were defined in the center and at the eastern, southern, western, and northern periphery of the disc, labeled by the numbers 1–5, respectively (see Figure 2).

For each trial, participants were cued about the next reach target by a small red disc near the target block. The cue was shown for 1 s, and participants had to direct their gaze at the respective number. In case the robot hand shadowed the cued location, or the participants inadvertently missed the cue, they were instructed to gaze at the target that they believed had been cued. After each cue, the disc was flickering for 2 s, and EEG readings were recorded during this interval. When the stimulation interval was over, participants reached to the cued target location and tapped on the top of the wooden block. They had another 2 s time for this action, including moving the hand back to the rest position in front of the abdomen. Then the next trial started by cueing another, randomly selected target location. Ten trials per target were collected in the training session. The size of the flicker stimulus, its frequency, the total number of targets as well as the stimulation period all affect the BCI's classification accuracy, and we adjusted these parameters on the basis of a pilot experiment.

With the EEG data from each trial, a canonical correlation (CCA) with a sine and cosine reference signal at the flicker frequency was calculated, and the resulting correlation coefficients formed features for linear discriminant analysis (LDA). Data from the training session were used to train the classifier. In the three experimental conditions, EEG data were classified online after each trial, and the classifier output, i.e., the number that the human gazed at, was sent to the robot controller.

### 2.5. Robot trajectory generation

#### 2.5.1. Target selection

The target location for the robot to reach was selected online based on the predicted reach goal of the human. If the operator started moving during a reaching motion of the robot, the robot's current target was discarded, and a smooth trajectory was generated as described below. To simplify the target selection process, we manually encoded geometric dependencies between targets by the set of conditions that is illustrated in Figure 2. Essentially, these rules prevented the robot from trying to reach a target which is physically blocked by the human arm during the reaching motion. The robot still reached for each target in order, but skipped the ones occupied by the current human motion prediction. Note that even when an invalid target was selected for the robot due to an erroneous prediction of the human's reach target, collisions still were avoided because the trajectory optimization, relying on the arm tracking data, made the robot remain in a local minimum until the human unblocked the target or the target changed.

#### 2.5.2. Trajectory optimization algorithm

With goal positions and human arm pose information, collision-free, and goal-directed trajectories were generated by the trajectory optimization algorithm in a Model Predictive Control (MPC) style. MPC adjusts the control variables to minimize an objective function under defined constraints (Morari et al., 1988). *l*_1_ is the robot first finger tip's (FFT) pose loss and is used to drive the FFT pose (*x*_*k*_, *rot*_*k*_) to the desired pose (*x*_*des*_, *rot*_*des*_) in Cartesian space. *rot*_*des*_ is set to prevent the robot from shadowing other target objects on the table from the viewpoint of the operator. The constant *c* is used to generate more fluent and consistent robot trajectories, and *N* is the number of robot trajectory waypoints.


(1) $$\begin{array}{l} l_{1}=\overset{N}{\underset{k=1}{\sum }}min\left(c,||x_{k}-x_{des}||\right)+||rot_{k}-rot_{des}|| \end{array}$$

The generated trajectories should satisfy the robot's kino-dynamic constraints:


(2) $$\underline{q}\leq q_{k}\geq \bar{q}$$

(3) $$\underline{\dot{q}}\leq \dot{q_{k}}\geq \bar{\dot{q}}$$

(4) $$\underline{\ddot{q}}\leq \overset{¨}{q_{k}}\geq \bar{\ddot{q}}$$

We modulated the robot's velocity limits in the BCI+VCV condition by the vigilance level as $-\underline{\dot{q}}=\bar{\dot{q}}=(1+0.3\;vigilance)V_{max}$. Vigilance levels were clipped to [−1, 1], resulting in a speed limit between 0.7*V*_*max*_ and 1.3*V*_*max*_. In contrast, the velocity limit was fixed at 0.7*V*_*max*_ in the BCI and AT conditions. Hence the robot arm was moving generally faster in the BCI+VCV condition than in the other two conditions.

In order to get smooth movements, velocity and acceleration were also regulated. *c*_1_ and *c*_2_ were weights of the loss function.


(5) $$\begin{array}{l} l_{2}=\overset{N}{\underset{k=1}{\sum }}c_{1}{\dot{q_{k}}}^{2}+c_{2}{\ddot{q_{k}}}^{2} \end{array}$$

**Figure 3:**
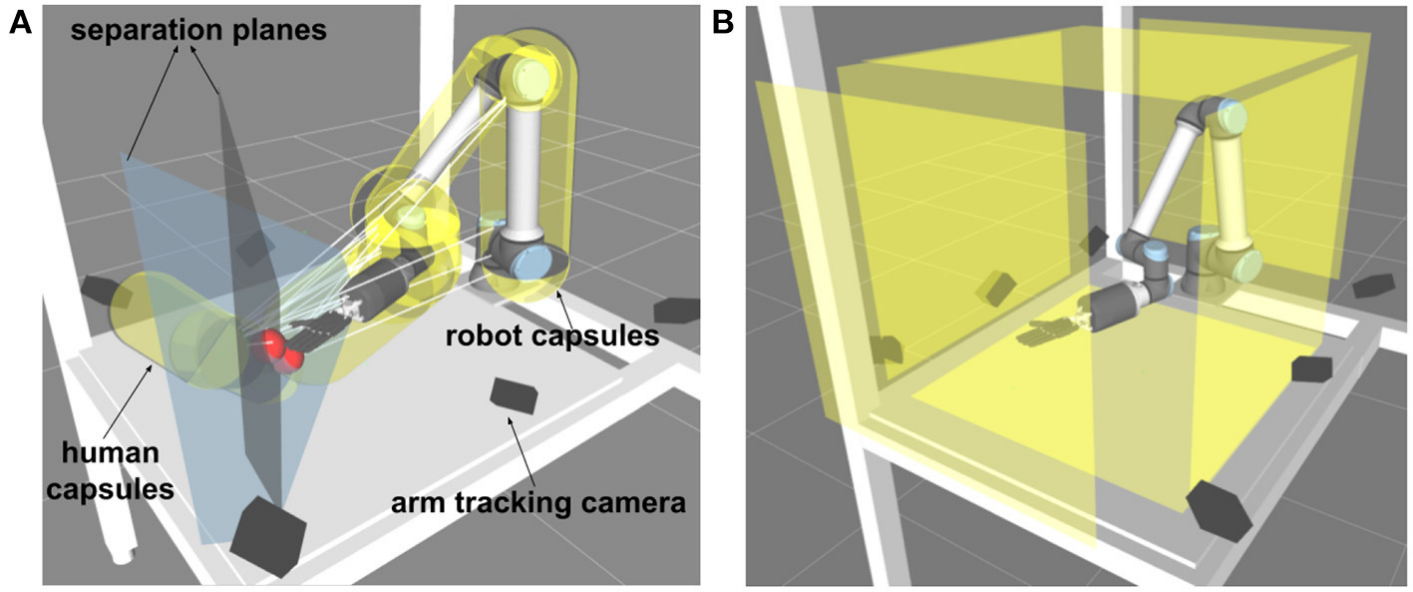
Geometric model of the work cell. In this view, the human stands on the left side of the table with only the right arm reaching into the shared workspace. **(A)** We use capsules (yellow) as geometric primitives for each part of the robot and the operator arm (palm, wrist, lower arm, upper arm). Trajectory planning and collision avoidance were based on the pairwise human-robot capsule distances, which can be calculated efficiently. Also shown are two separating planes (blue) with their origins (red) on the midpoint between the two closest pairs (human-hand and -palm vs. robot palm) of capsules. **(B)** Static boundaries of the robot workspace; no part of the robot was allowed outside of the cuboid defined by the six planes.

To avoid collisions between the robot's and the operator's arm, we modeled both arms by capsule-shaped collision objects. Capsules with a radius ${\left(r_{a,i}\right)}_{i=1}^{4}$ were created between all connected joints of the operator's arm, and capsules with a radius ${\left(r_{r,j}\right)}_{j=1}^{6}$ were created between all connected robot joints. Then the two closest points between every paired robot-operator arm capsules were calculated, and the segment line connecting these two points was the normal of the separation plane *N*_*i,j*_ which separated the two capsules *i* and *j* (see Figure 3). The positions of the arm joints and the robot joints in Cartesian space were denoted by ${\left(P_{a,i}\right)}_{i=1}^{4}$, ${\left(P_{r,j}\right)}_{j=1}^{6}$, respectively. Then soft constraints were used to avoid collisions between the robot's and the operator's arms.


(6) $$\begin{array}{l} l_{3}=\overset{4}{\underset{i=1}{\sum }}\overset{6}{\underset{j=1}{\sum }}min\left(0,{\left(N_{i,j}^{T}\left(P_{a,i}-P_{r,j}\right)-r_{a,i}-r_{r,j}-d\right)}^{2}\right) \end{array}$$

Six boundary planes ${\left(N_{b,m}\right)}_{m=1}^{6}$ were employed as hard constraints to restrict robot motion in the desired area as shown in Figure 3.


$$P_{r,k}^{T}N_{b,m}\leq 0,\forall \;k\in \left[1,N\right],m\in \left[1,6\right]$$

**Figure 4:**
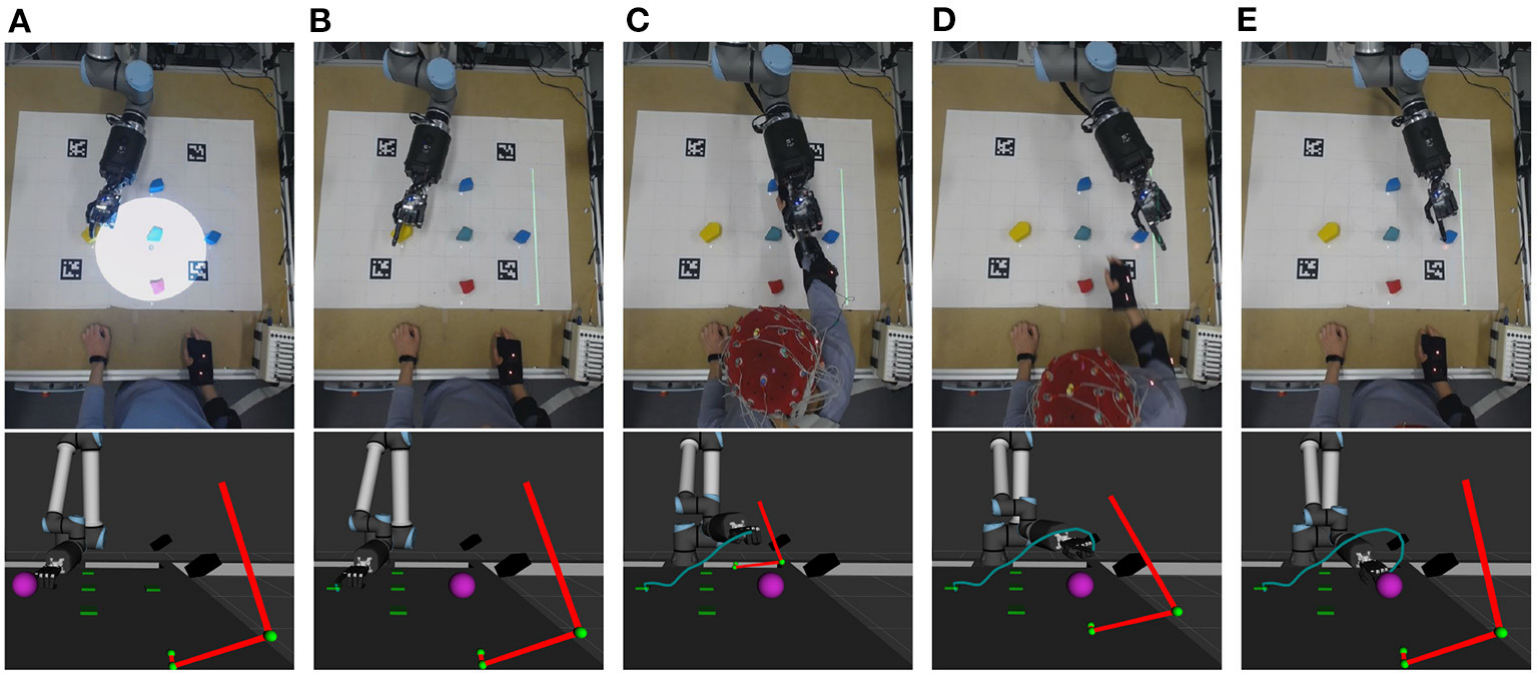
Example scenario for the robot arm trajectory generation based on the operator's movement prediction and arm tracking. The purple sphere is the goal position of the robot's first finger tip. Red cylinders visualize the current position of the operator's arm. The green curve represents the trajectory of the first finger tip. **(A)** The operator gazed at target object no. 5, and the robot touched object 4. EEG signals were collected in this state (white circle shows flicker stimulus). **(B)** The BCI predicted the operator will target object 5, and the robot controller adjusted its next goal from target object 5 to target object 2 after touching target object 4. **(C–E)** The operator touched target object 5, then returned to the rest position. A collision-free robot trajectory was generated by the proposed trajectory optimization method.

During the experiments, the trajectory optimizer-related parameters were set to the values in Table 1. The robot trajectory was optimized at 10 Hz with the latest operator intention prediction and vigilance estimation results. As the foundation of our implementation, a primal-dual interior-point solver, proposed in Ruppel and Zhang (2020), was used to solve the optimization problem. One example trajectory during the experiment is shown in Figure 4.

**Table 1:** Trajectory optimizer parameters.

**Parameter**	**Value**
Radii of capsules	0.1*m*
Joint velocity limit *V*_*max*_	0.02*rad*/*s*
Joint acceleration limit	1*rad*/*s*^2^
Robot trajectory length *N*	3
Weights *c*_1_, *c*_2_	1
Safety offset *d*	0*m*
Constant value *c*	0.2*m*

#### 2.5.3. Minimum distance analysis

During the experiment, operator arm, robot joint trajectories, and cue signals were recorded synchronously. The data were then segmented into trials. Afterwards, the minimum distance (MD) per trial between the human arm and robot limbs was calculated based on the method described in the trajectory optimization section above. MD values were used for HRC safety assessment.

## 3. Results

### 3.1. HRC performance

**Figure 5:**
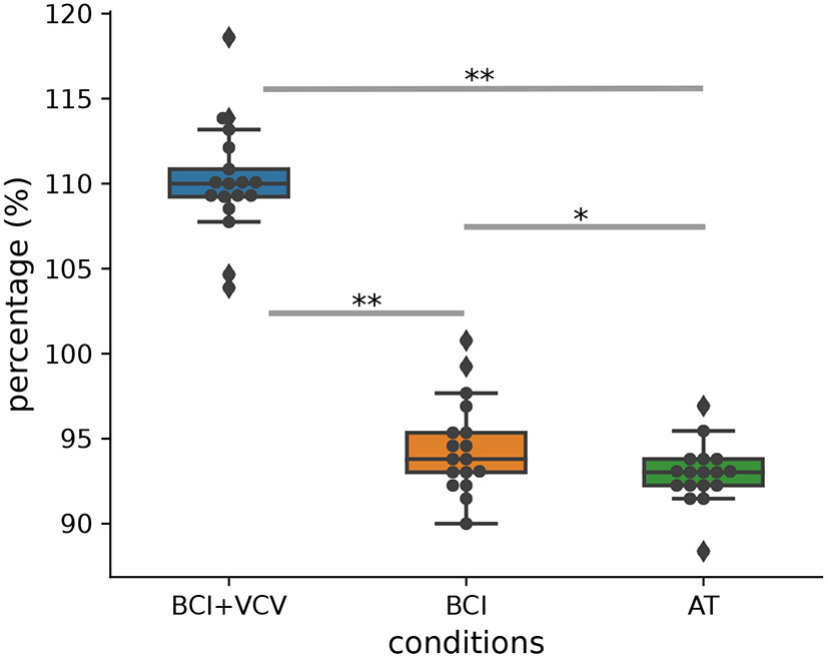
Robot performance relative to the training session. The horizontal line in a box shows the performance median. The upper and lower lines of a box represent the upper and lower quartiles, respectively. The whiskers extend to points that lie within 1.5 interquartile ranges of the lower and upper quartile. ^*^p < 0.05, ^**^p < 0.001.

The main indicator for the robot's performance was the number of targets touched in a session. We considered the robot performance in the training session, when the operator could not interfere with the robot's actions, as the baseline and compared it with the performances in the BCI+VCV, BCI as well as AT sessions. We found that the mean performances in these three conditions were about 110.28, 95.04, and 92.76%, respectively (Figure 5). Paired *t*-tests suggest significant differences between the conditions [BCI+VCV vs. BCI: *p* = 1.67*e*^−13^, BCI+VCV vs. AT: *p* = 1.95*e*^−11^, BCI vs. AT: *p* = 0.039, Lilliefors' test (Conover, 1999) for normal distribution]. The result indicates that considering the operator's action for planning the robot's movements degrades performance, but that this negative impact can be compensated by adjusting the robot's velocity according to the attentional state of the operator.

### 3.2. Safety distance analysis

In an HRC scenario, robot performance should not be the only performance indicator though. The safety of the interaction, evaluated by the minimum distance between the robot's and the operator's arm, should be kept above a safe limit.

**Figure 6:**
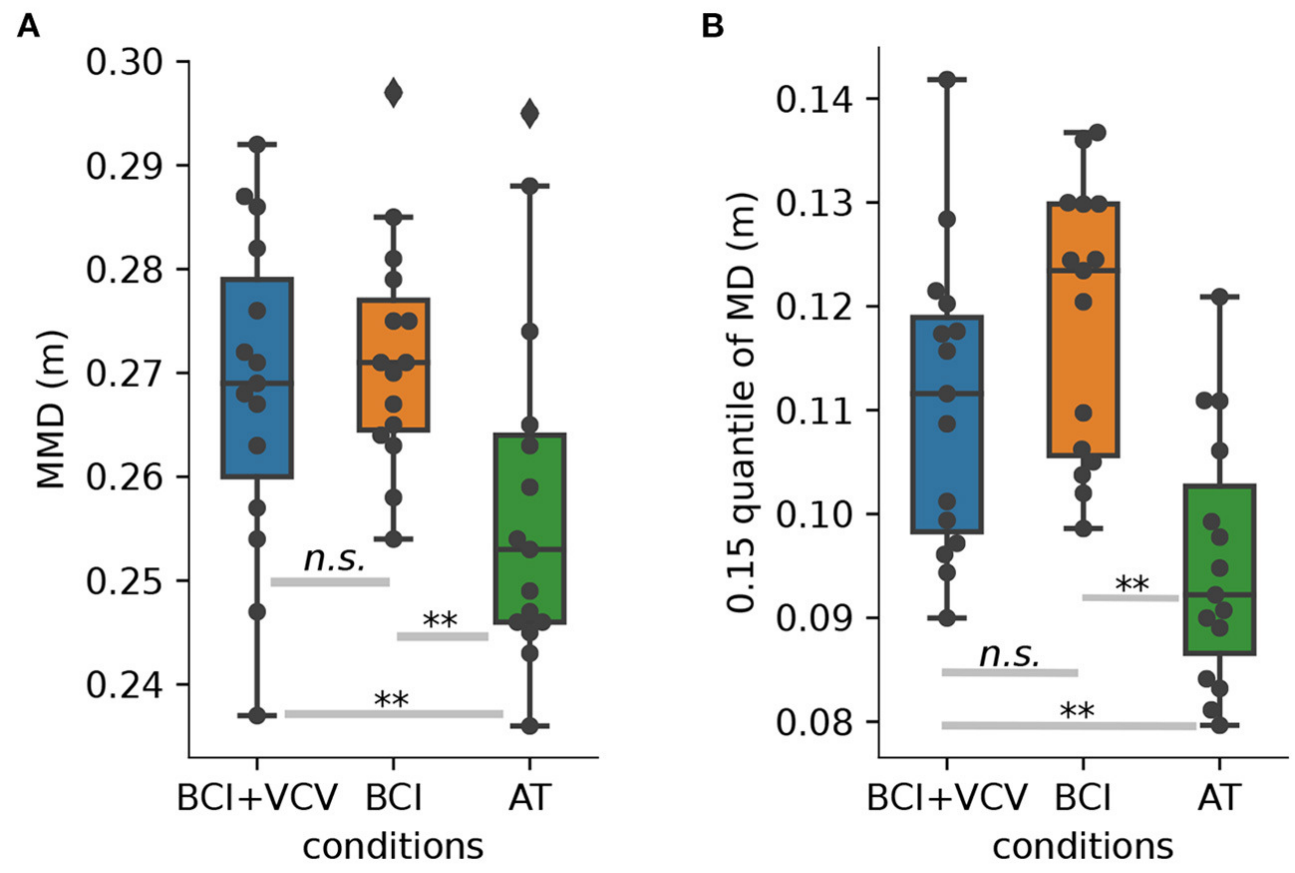
**(A)** MMD distribution per participant over sessions and t-test results for significance check. **(B)** Distribution about 0.15 quantile of MD distribution per participant over sessions and *t*-test results for significance check. ^**^p < 0.01. n.s., not significant where *p* > 0.05.

The distribution of the mean minimum distances (MMDs) across participants shows that the BCI condition resulted in the largest safety distances (Figure 6A). Despite the higher robot arm velocity, safety distances were only marginally smaller in the BCI+VCV condition (*p* > 0.49). In contrast, MMDs were substantially smaller in the AT condition (both *p* < 0.01).

In order to further investigate whether the BCI+VCV or BCI methods could improve HRC safety, we calculated the 0.15 quantile of the MMD per participant per session. The distributions over participants are shown in Figure 6B. The 0.15 quantile of the BCI condition seems to be somewhat higher than the BCI+VCV condition (*p* = 0.058) but substantially higher than the AT condition on average (*p* < 0.01).

The trajectory optimization algorithm was instructed to ensure that the distance between the operator's and robot's arm was kept above 20 cm. Individual samples in Figure 6 indicate, however, that the distance fell below this threshold in some trials. To elucidate whether the sub-threshold distances were caused by fast movements of the operator or the ineffectiveness of the trajectory optimization algorithm, we selected five participants randomly and analyzed the relative motions between their and the robot's arm. We only considered situations where the minimum distance was < 20 cm. The minimum distances (*d*_*t*_), velocities (*v*_*Pt*_, *v*_*Rt*_) as well as accelerations (*a*_*Pt*_, *a*_*Rt*_) of the closest points between the operator and the robot were calculated per time step *t*. All of the time steps were divided into three groups: (1) the robot arm was moving away (MA) from the operator's arm (*d*_*t*+1_ > *d*_*t*_); (2) the robot was braking (BR, *d*_*t*+1_ < *d*_*t*_, but the angles between *v*_*Pt*_ and *v*_*Rt*_, *v*_*Pt*_, *a*_*Rt*_ were larger than 90°); (3) the robot failed to avoid the operator's arm (FA, time step is neither MA nor BR). For this experiment, the robot was set to not consider the operator's movements when it was touching the block. However, the operator's target could be the same as the one the robot was touching already. This resulted in several FA cases where robot velocities were zero.

**Figure 7:**
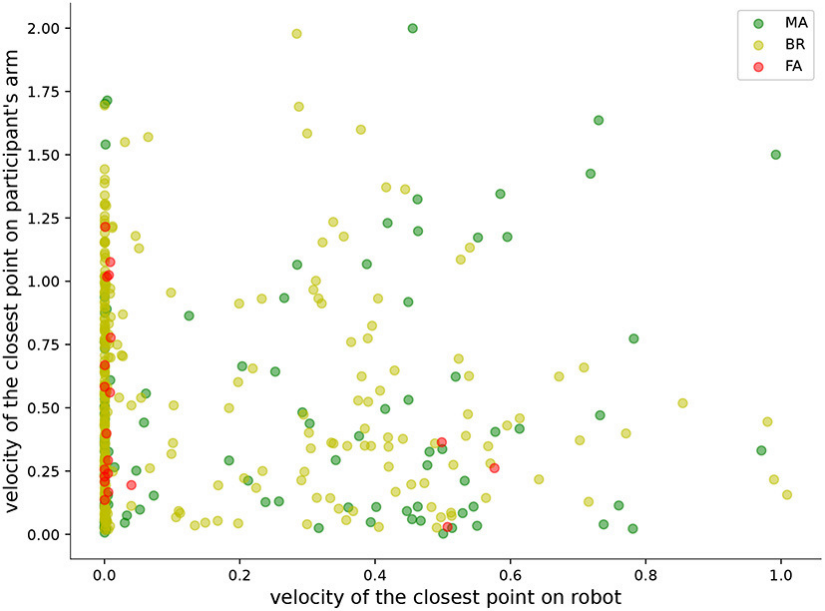
Velocities of the pairwise closest points between the operator's and the robot's arm. MA, robot was moving away; BR, robot was braking; FA, robot failed to avoid operator arm.

Looking at the distribution of collision situations in Figure 7 suggests that almost all of them resulted from swift movements of the operator, and the robot could not move quickly enough to keep the desired safety distance because of its dynamic limitations.

### 3.3. Target prediction accuracy and vigilance

**Figure 8:**
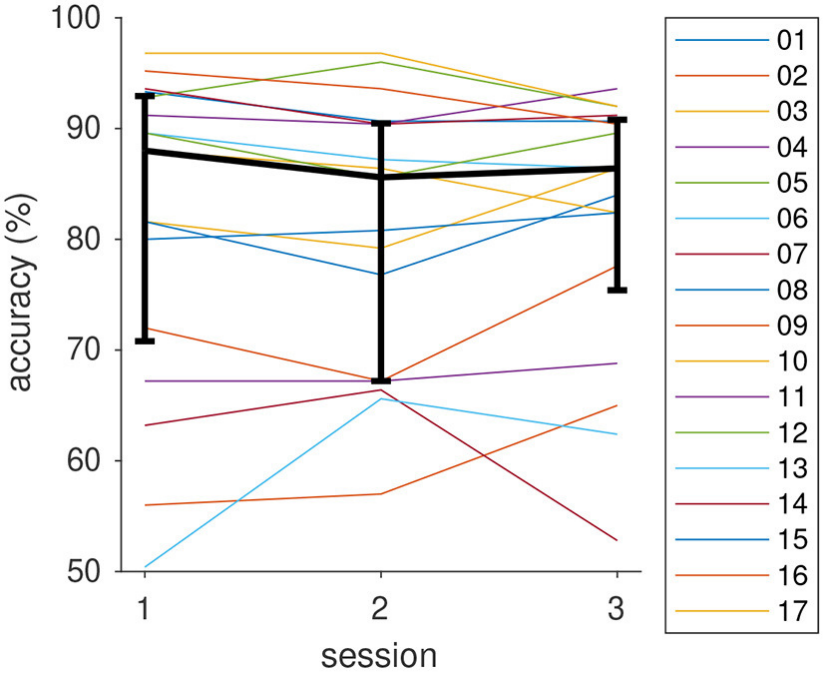
BCI classification accuracy of the participants in the three sessions. Thick black curve is the median, error bars show the 25 and 75%, quantiles.

In the first session after training, the reach target prediction achieved a median accuracy of 88% across all participants. The performance remained stable in the two subsequent sessions (85.6, 86.4%, see Figure 8). Pairwise Kruskal–Wallis tests support this observation (all *p* > 0.93). Whereas the BCI achieved a prediction accuracy of 80% or better for the majority of participants, accuracies can be as low as 50% for individual operators. The observation that the performance of these individuals remains below 80% for all conditions suggests that this reflects individual traits rather than concentration lapses, novelty, or training effects etc.

**Figure 9:**
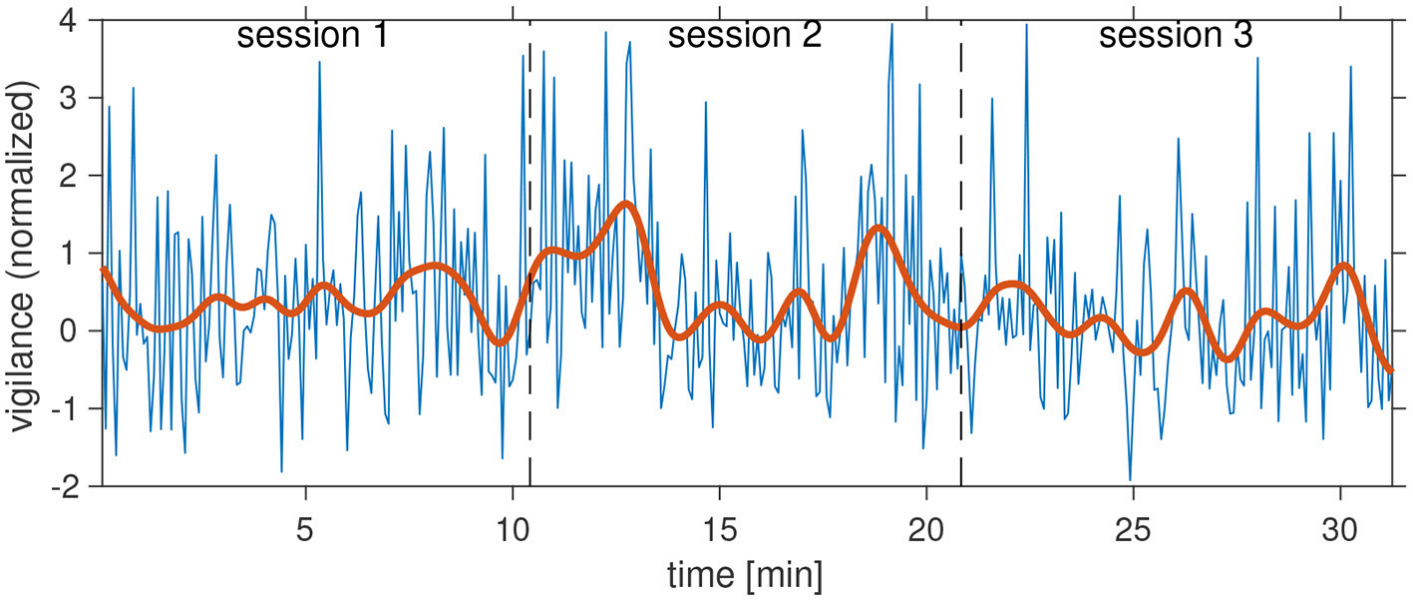
Vigilance time course of an individual participant (08). Blue curve: Vigilance (SSVEP SNR) for each trial, red curve: low-pass filtered vigilance time course (0.01 Hz cut-off).

In Figure 9, we show an example for the time course of the vigilance over the three application sessions. As expected, vigilance varies between trials. However, there are clearly episodes in which vigilance tended to have higher values and episodes in which lower values dominated. In the example, vigilance decreased at the end of each session, and values were generally lower in session 3. Episodes of high vigilance can be noted at the beginning and toward the end of session 2 with a pronounced drop in between.

**Figure 10:**
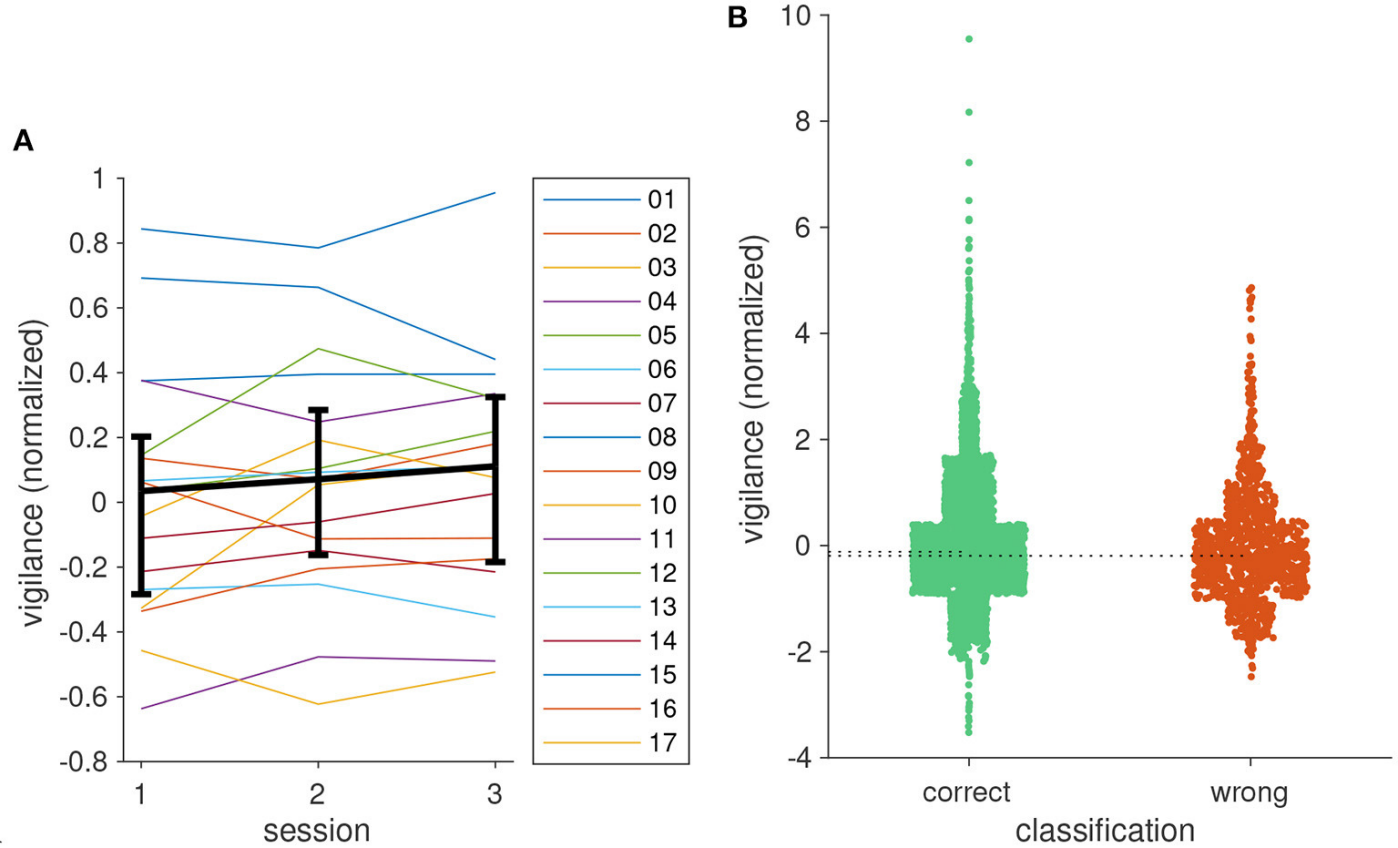
**(A)** Median vigilance levels for the three sessions. Error bars show the 25 and 75% quantiles. **(B)** Distribution of vigilance values for trials with correct and incorrect classification results. Width of a scatter block corresponds to the number of samples in the interval marked by its height. Dotted lines indicate medians of the two distributions.

Averaging vigilance values across trials and participants in Figure 10A shows that, like for BCI performance, participants were able to keep up attention over the course of the experiment. Pairwise Kruskal–Wallis tests likewise gave no indication for systematic changes of attention levels between sessions (all *p* > 0.79).

We were interested in the question whether vigilance had an effect on classification accuracy. To this end, we analyzed the distribution of vigilance values conditioned on the classification result. The plot in Figure 10B shows that both distributions largely overlap. Nevertheless, the median of vigilance values in trials with classification errors was slightly lower than in trials where the classification was correct (−0.1165 vs. −0.1932, *p* = 0.043, Kruskal–Wallis test). One problem of this analysis is that vigilance is not the only factor that affects classification accuracy. The robot arm casting a shadow on the flicker stimulus or hiding target locations as well as participants not looking at the cued target likely result in classification errors. This may explain the high values in the distribution of vigilance values for wrong classifications in Figure 10B, leading to an underestimation of the true difference of the medians. Hence, low vigilance likely impedes classification accuracy, but it nonetheless seems fairly robust against attention lapses.

**Figure 11:**
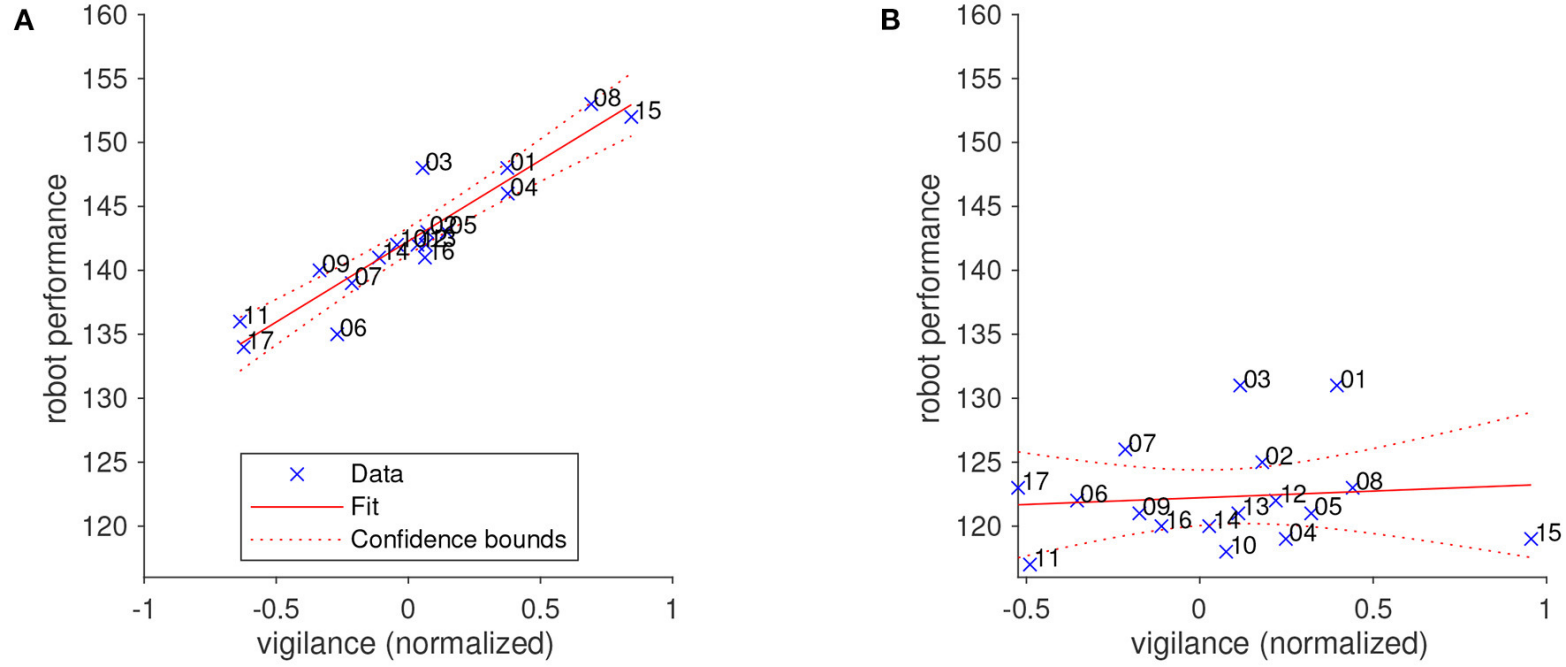
The relationship between human vigilance and robot performance fitted by a linear model. **(A)** The robot's velocity is controlled by the operator's vigilance (BCI+VCV condition). **(B)** Robot velocity is not modulated by vigilance (BCI condition). Numbers at data points indicate participant number.

To verify that vigilance was the driving factor in the performance of the robot in the BCI+VCV condition, we analyzed the relationship between the average normalized vigilance of each participant and the robot performance in the BCI+VCV session. Figure 11 shows that a linear model is an adequate fit for this relationship (*p* = 5.74*e*^−8^). We did not observe a similar relationship between BCI classification accuracy and robot performance, corroborating the finding that target prediction and vigilance are independent information channels that are provided by the BCI.

## 4. Discussion

Robots and humans excel at different tasks, and HRC is becoming a critical concept for combining their respective expert skills (Castro et al., 2021). Robots moving at high velocities could improve HRC efficiency, but they are liable to compromise safety. Reliably predicting human intention may come as a solution to this problem. In robotics, many approaches for intention prediction employ motion tracking of the human body or parts thereof (Pérez-D'Arpino and Shah, 2015; Luo et al., 2018; Ravichandar et al., 2018; Park et al., 2019; Trick et al., 2019; Cheng et al., 2020; Cooper et al., 2020). They have in common that accurate predictions can be made only after the movement of the human partner commenced. When the potential targets are located closely to each other, the initial segments of the hand's motion trajectories will be similar; therefore, reliable predictions can be made only after a substantial part of the reach movement has been executed.

In order to predict the human's intention earlier, EEG-based methods have been investigated in previous studies (Shafiul Hasan et al., 2020). For example, the Bereitschafts-potential, a conspicuous deflection in the EEG signal about 500 ms before movement onset, has been used to predict when the human will move the arms and which kind of grasp action will be executed (Buerkle et al., 2021; Xu et al., 2021).

As hand and eye are coordinated during human action execution, analyzing the gaze direction may allow predicting impending movements early on. Instead of using optical eye-tracking devices, we here used a BCI to demonstrate that this technology enables gathering information about the cognitive state of the operator in addition to the gaze direction. The majority of studies about BCI methods for robotics focus on the development of BCI components or evaluate the BCI technology in simulation (Buerkle et al., 2021). Our study extends previous investigations and demonstrates an integrated system for a closed-loop BCI-controlled HRC scenario.

### 4.1. BCI can improve HRC efficiency and enhance safety

We evaluated our approach in a study in which the participants performed a pick-and-place task together with a robot arm in a narrow shared workspace. Our system monitored the participants' vigilance in the EEG signals and increased or reduced the robot's velocity depending on whether vigilance was high or low. Our results show that BCI-based intention prediction, in particular in combination with vigilance-modulated velocity, can improve HRC performance and safety compared to a motion-tracking-based approach. In especial, the higher performance with the vigilance-controlled velocity did not degrade the HRC safety level indexed by the minimum distance between the operator's and the human's arm.

Since the BCI allows the robot to know the reach target before the human actually deploys the action, it has sufficient time for adjusting its task position accordingly. This leads to the performance increase that we observed in the two BCI-enabled conditions compared to the AT condition, in which predictions relied solely on motion data. By evaluating the vigilance, we could adjust the robot velocity according to the level of the operator's alertness. As the result of this strategy, the robot performance improved further in the BCI+VCV condition as shown in Figure 5.

The performance of the BCI classifier, however, varies across participants and remains below 80% for some of them. This distribution is a well-known property of every BCI paradigm and has been termed “BCI illiteracy” (Allison et al., 2010). Lower BCI performance may result from a mismatch between the stimulation parameters, which were fixed for all participants, and the individual response properties. By optimizing flicker frequency, stimulus size and stimulation duration for each participant, accuracy of the intention prediction could be improved. In case such a parameter optimization does not yield the expected improvement, the system can still rely on motion-tracking data for predicting the target location of a reach movement.

Another important advantage of using BCI for HRC is safety improvement. MPCs have been studied for almost 40 years. They can generate optimal robot control commands in a short time horizon in the future by solving an optimization problem with various constraints and objective functions (Morari et al., 1988). A prerequisite of MPC is that changes in the environment can be predicted in advance. Several studies have shown that the combination of human motion prediction and MPC can enhance safety in HRC (Li and Shah, 2019; Park et al., 2019; Zhao et al., 2020). Therefore, we employed MPC in our approach and modeled, for simplicity, the predicted operator's reach trajectory by a cylinder which connected the human's palm joint and the predicted target position. This method accounts for human arm movements being very fast and the duration of reaching movements being very short. Hence, early intention prediction together with the proposed MPC-style trajectory optimization algorithm could also enhance the HRC safety in this study. For more complex optimization problems, e.g., with non-convex constraints or objective functions, the learning-based methods could also be used, like in Su et al. (2020), to improve the computation speed.

One more interesting result we found is that the BCI+VCV method, in spite of higher average robot arm velocities as described in the trajectory optimization algorithm part, maintained similar safety metrics like the BCI method in which the velocity was fixed. Typically, faster robot movements increase the danger of collisions in HRC (Buerkle et al., 2021). If the robot controller is designed to account for the operator's attentional state, higher velocities may be tolerable without reducing safety margins.

## 5. Conclusion and future work

In conclusion, our findings suggest that the proposed BCI+VCV strategy together with the MPC-style trajectory optimization algorithm could improve HRC efficiency and safety at the same time. Performance could be further improved with a better operator trajectory prediction module using, e.g., an artificial neural network. Thus, the robot could adjust its trajectories more precisely to ensure operator safety.

For future work, one interesting direction is to search for new methods to predict human intentions from EEG signals with shorter latencies. For example, in this work, the operator had to gaze at the intended target position for 2 s in order to collect enough EEG data for a reliable classification. By reducing the amount of data to a few hundred milliseconds, human intention could be predicted faster and thus HRC performance would increase further.

Another direction we propose to investigate are alternative strategies to modulate the robot velocity according to the alertness of the operator. For example, one could use a neural network instead of the linear relation here to capture more suitable and likely more complex relationships. The input of the network could be EEG signals or vigilance estimates, and the network would output speed limits for the robot. This network could be trained together with the robot in a virtual reality environment. It would also be interesting to develop methods for fusing the multi-modal information from EEG, electromyography (EMG), and motion tracking for faster and more detailed intention and movement prediction.

## Data availability statement

The raw data supporting the conclusions of this article will be made available by the authors upon request, without undue reservation.

## Ethics statement

The studies involving human participants were reviewed and approved by the Ethics Committee of the Medical Association of the city of Hamburg and the Ethics Commission of the Department of Informatics, Universität Hamburg. The participants provided their written informed consent to participate in this study.

## Author contributions

JL and AM were responsible for providing the study idea, experiment design, experiment execution, result analyses, and manuscript draft. MG and PR gave supports during the experiment execution. All authors provided valuable feedback on the manuscript.

## Funding

This work was funded by the German Research Foundation (DFG) through project TRR169/B1/B5/Z2/Z3. This work was also partially funded by China Scholarship Council (CSC).

## Conflict of interest

The authors declare that the research was conducted in the absence of any commercial or financial relationships that could be construed as a potential conflict of interest.

## Publisher's note

All claims expressed in this article are solely those of the authors and do not necessarily represent those of their affiliated organizations, or those of the publisher, the editors and the reviewers. Any product that may be evaluated in this article, or claim that may be made by its manufacturer, is not guaranteed or endorsed by the publisher.
